# Cost-effectiveness in unstable economies: the case of sacubitril/valsartan in heart failure with reduced ejection fraction in Argentina

**DOI:** 10.1186/s13561-023-00427-w

**Published:** 2023-02-18

**Authors:** Mariano A. Giorgi, Carlos P. Boissonnet, Paula Soledad Luque, Jimena Piastrella, Carlos Porley, Fernanda Ditata, Sergio Volman

**Affiliations:** 1 Health Economics and Technology Assessment Unit. Centro de Educación Médica e Investigaciones Clínicas “Norberto Quirno” (CEMIC), Instituto Universitario CEMIC (IUC), Buenos Aires, Argentina; 2 Cardiology Section. Department of Internal Medicine. Centro de Educación Médica e Investigaciones Clínicas, Norberto Quirno” (CEMIC), Buenos Aires, Argentina; 3 Associate Professor of Pharmacology, Instituto Universitario CEMIC (IUC), Buenos Aires, Argentina; 4 Novartis Pharmaceutilcals, Basel, Switzerland; 5 Piastrella Worked at Novartis at the Time This Research Was Conducted, Basel, Switzerland

**Keywords:** Cost-effectiveness, Heart failure, Discount rate

## Abstract

**Background:**

Sacubitril/valsartan (an Angiotensin receptor-neprilysin inhibitor—ARNI) is one of the cornerstones in the management of patients with heart failure with reduced ejection fraction (HFrEF) having demonstrated significant reductions in both mortality and hospitalisations as compared with enalapril. It proved to be a cost-effective treatment in many countries with stable economies. In Argentina, a country with chronic financial instability and a fragmented health care system, the estimation of its cost-effectiveness requires to consider local financial data.

**Objectives:**

To estimate the cost-effectiveness of sacubitril/valsartan in HFrEF in Argentina.

**Methods:**

We populated an Excel-based cost-effectiveness model, previously validated, using inputs from the pivotal phase-3 PARADIGM-HF trial and from local sources. As the main problem to consider was the financial instability, we adopted a differential approach to cost discounting based on the opportunity cost of capital. Thus, a discount rate for costs were set at 31.6%, using the BADLAR rate published by the Central Bank of Argentina. Discount for effects were set at 5% as is the current practice. Costs were expressed in Argentinian pesos (ARS). We used the perspective for both the social security and private payers at a 30-year horizon. The primary analysis was the incremental cost-effectiveness ratio (ICER) versus enalapril, the previous standard of care. Alternative scenarios performed included a 5% cost discount rate and 3 a 5-year horizon (as is usually used).

**Results:**

In Argentina the cost-per quality adjusted life-year (QALY) gained for sacubitril/valsartan versus enalapril was 391,158 ARS and 376,665 ARS for a social security and a private payer, respectively, at a 30- year horizon. These ICERs were under the cost- effectiveness threshold of 520,405.79 ARS (1 Gross domestic product (GDP) per capita) suggested by Argentinian health technology assessment bodies. Probabilistic sensitivity analysis showed an acceptability of sacubitril/valsartan as a cost-effective alternative of 86.40% and 88.25% for social security and private payers, respectively.

**Conclusion:**

Sacubitril/valsartan is a cost-effective treatment in HFrEF using local inputs that considered the financial instability. For both payers considered the cost per QALY gained are under the cost-effectiveness threshold considered.

**Supplementary Information:**

The online version contains supplementary material available at 10.1186/s13561-023-00427-w.

## Background

Heart failure (HF) is a clinical syndrome that represents a common cause of hospitalization and death worldwide. At a global level, its prevalence has increased in parallel with the aging of the population and the presence of cardiovascular risk factors, especially hypertension and diabetes mellitus [1]. HF classification is based on the measurement of the left-ventricular ejection fraction (LVEF) and the type with reduced ejection fraction (HFrEF) is the one with the highest in-hospital mortality despite multiple drugs and devices available [2]. The results from the PARADIGM-HF trial demonstrated that, in patients with HFrEF, sacubitril/valsartan (an Angiotensin receptor-neprilysin inhibitor – ARNI) compared to enalapril reduced the primary endpoint of death from cardiovascular causes or hospitalization for heart failure (hazard ratio confidence interval (CI) 0.80 (0.73 to 0.87); *P* < 0.001) and improved quality of life [3]. The drug was also included as a standard of care in recent clinical guidelines [4, 5]. Besides, the added value of sacubitril/valsartan led to its adoption as a cost-effective treatment by several health technology assessment agencies [6, 7].

Heart failure information in Latin America is scarce. The estimated prevalence of HF is 0.13 to 2.74% [8] but there are no epidemiological data about HFrEF. Data from HFrEF´s clinical trials revealed that clinical characteristics and outcomes are quite similar to those observed in Western countries [9] and it is reasonable to assume that sacubitril/valsartan could be deemed as effective as in other world´s regions. However, Latin-America includes countries with a heterogeneous mix of races and socio-economic environments that could affect the cost- effectiveness of therapeutic interventions. One of the most important issues in this regard is the financial instability in countries like Argentina, with return of investment and inflation rates that are generally higher than those observed in Western countries [10]**.** Drugs, like sacubitril/valsartan, that could reduce both mortality and hospitalizations and, consequently the high costs of HFrEF [11, 12] should also demonstrate its added value for the proposed price to be included in the constrained Argentinian health system, which consist in three main payers (public, private and social security). In Argentina it has been considered that sacubitril/valsartan would imply a high budgetary impact for the public health system [13]. However, this document did not assess the cost-effectiveness nor contemplate the possible influence of the unstable local financial variables. Considering that the recommendations on transferability of health technology assessment data highlights the importance of using local and sound inputs [14], the unstable financial environment of Argentina raises the question about which is the most appropriate discount rate for costs. The usual practice of using a cost´ discount rate based on the social time preference (set between 3 to 5% in most countries) [15] might be inappropriate and considering other economically robust principles, as the opportunity cost of capital [16], could provide a discount rate more closely to local financial conditions. Therefore, our aim was to estimate the cost-effectiveness on sacubitril/valsartan in HFrEF in Argentina using local financial inputs for both the social security and private health systems in a long-term scenario (30 years). Furthermore, we also aimed to explore the cost-effectiveness in shorter-term horizons at 3 and 5 years and using more traditional cost discount rates.

## Methods

A Microsoft Excel based model, property of the sacubitril/valsartan owner (Novartis Pharmaceuticals) was used.

The model is structured as a two-state Markov model (with health states “alive” and “dead”), with regression models used to predict events and outcomes such as mortality, hospitalisations, adverse events, and health-related quality of life over the time horizon of the model, based on patient characteristics and treatment received. This same model was employed by the company in their submission to the National Institute for Health Care and Excellence (NICE) in the UK [6] and described in detail elsewhere [17]. It considered a primary analysis comparing sacubitril/valsartan versus enalapril in patients with HFrEF based on the efficacy and adverse events rates reported in the PARADIGM-HF data [3]. A secondary analysis included a comparison against valsartan using efficacy and adverse events data from a network meta-analysis about drug therapies in chronic HF [18].

### Model inputs

Population characteristics were obtained from a subset of 1433 patients from Latin-American countries included PARADIGM-HF study [3].

Health states and transition probabilities were obtained from the PARADIGM-HF study and from a sacubitril/valsartan cost-effectiveness model [3, 17, 19]. All cause and cardiovascular mortality were derived from the PARADIGM-HF trial adjusted to the national statistics [20] and annualized using a smoothing method [21]. Hospitalisation rates were obtained from the PARADIGM-HF study [3], as well as the incidences of every pre-specified safety event in the trial, as follows [3]: hypotension and angioedema (more frequent with sacubitril-valsartan), and elevated serum creatinine, elevated serum potassium and cough (more frequent with the comparator enalapril). For the secondary analysis with valsartan (an angiotensin receptor antagonist – ARB), in the absence of data it was assumed that adverse event rates were equivalent between sacubitril/valsartan and ARB, since sacubitril/valsartan contains the ARB valsartan [17].

Because there were no local utilities data published, we used derived values from the EQ-5D questionnaire from the PARADIGM-HF study [3, 19]. Utilities were calculated based on a mixed-effects model based on EQ-5D scores reported at baseline and over time during the PARADIGM-HF trial [3, 19].

Resource utilization inputs, including HF in-hospital and ambulatory care management were obtained from local studies reporting real-world data [22, 23].

The perspective of both the social security and private health care payers were adopted. Costs were expressed in Argentinian pesos (ARS) and updated to December 30^th^, 2020. For reference, at this date 1 USD equalled 89 ARS. Hospitalization costs were obtained and updated from a local study [12]. Drug prices were obtained from public access database and adjusted to fit the payer´s perspective [24]. The price for sacubitril/valsartan was provided by Novartis.

In order to follow the transferability recommendations stated by ISPOR [14] the model was initially informed with local inputs. For that purpose, a systematic literature review was conducted searching for available epidemiological and clinical data. When local data was not available, model inputs were obtained from regional or international published sources. Inputs and sources used to inform the model were depicted in Tables 1 and 2. Economic analyses must apply a discount rate to costs and results, which must consider the effect of the passage of time on them. Future costs and benefits must be discounted from their value at the present time, using a standard discount rate, when the time universe of analysis is greater than 1 year. A 5% discount rate for effectiveness was initially considered as it is usually applied in health economic analysis. However, based on the aforementioned non-transferability principle [14] we adopted a differential approach for discounting costs because it should realize local opportunity cost. Therefore, we chose a return of investment rate intended to reflect the domestic conditions of the financial system more closely [25, 26] and the costs´ discount rate was set at 31.64%, the 12- month average for the BADLAR rate, a borrowing investment rate reported by the Central Bank of Argentina [10]. BADLAR is the name given to the interest rate for fixed-term deposits over one million pesos, from 30 to 35 days. The BADLAR, allows to capture the economic, financial and exchange instability that Argentina suffers. It is a variable rate that is calculated daily by the Central Bank of the Argentine Republic (BCRA), based on a sample of rates used in the Autonomous City of Buenos Aires and Greater Buenos Aires, it also takes into account the variation in value of the Leliq (BCRA liquidity bills) used to set/anchor inflation [10].

**Table 1:** General inputs used in the model

Input	value	comment	source
**Patient characteristics**		patients from Latin-American Countries randomised in the PARADIGM-HF	3
* Mean age (years)*	63		3
* Female %*	27.30%		3
* NYHA I %*	7.5%		3
* NYHA II %*	81.6%		3
* NYHA III %*	10.9%		3
* NYHA IV %*	0.0%		3
* LVEF %*	28.3		3
* Ischaemic aetiology %*	43.1%		3
* Previously hospitalised for HF %*	53.9%		3
* Mean SBP (mmHg)*	118.6		3
* Mean heart rate (bpm)*	70.6		3
* Mean eGFR (mL/min/1.73m2)*	68.7		3
* Prior ACEi use*	67.1%		3
* Prior ARB use*	33.3%		3
* Beta blocker use*	92.3%		3
* Mineralocorticoid receptor antagonist use*	64.5%		3
**Health states probabilities**			
* Hospitalization for HF*	2.69%	modelled monthly probability	3
* Cardiovascular Mortality*	0.58%	modelled monthly probability	3
**Utilities**			
* reduction for each year with HF*	-0.008		3, 19
basal utility in HF	0.807		3, 19
**Discount rate**			
* Outcomes*	5%	discount rate usually applied in HE studies in Argentina	
* Costs*	31.64%	according to the BADLAR rate, a return of investment rate considering the opportunity cost of capital	10
* Cost-Effectiveness Threshold*	≤ 1 GDP per capita	as suggested by the National Commission for Health Technology Assessment	27
**Time Horizon**	30 -years		19

**Table 2:** Cost inputs used in the model

input	Social security	Private	Source
**Costs of primary therapy**			
* Monthly cost of sacubitril/valsartan*	6028.38	6028.38	Novartis
* Monthly cost of enalapril*	91.92	91.92	24
* Monthly cost of valsartan*	390.78	390.78	24
**Costs of background therapy (monthly cost)**			
* Beta blockers*	194.59	194.59	24
* Mineralocorticoid receptor antagonists*	234.97	234.97	24
* Digoxin*	20.23	20.23	24
* Lipid lowering medications*	305.17	305.17	24
* Diuretics*	71.34	71.34	24
* Aspirin*	19.21	19.21	24
* Anticoagulants*	39.46	39.46	24
* ADP antagonists*	315.69	315.69	24
**Costs—HF Management (unit costs)**			
* GP emergency visits*	2495.50	4073.24	30
* GP visits*	464.81	873.17	30
* Cardiologist visits*	841.46	1318.90	30
* Cost per hospitalisation*	129,939.15	228,109.46	12

*Costs are expressed in Argentinan pesos (ARS). Exchange rate: 1USD* = *89 ARS*

Despite not being mandatory, the cost-effectiveness threshold in Argentina was recently set at ≤ 1 GDP per capita by the National Commission for Health Technology Assessment [27]. Considering a population of 45,376,763 inhabitants [28] 1 GDP per capita equalled to 520,405.79 ARS (5,801.62 USD) [29].

For the deterministic base case analysis both costs and effects were discounted at a 30-year time horizon following the original model settings and results were reported as the incremental cost-effectiveness ratio for quality-adjusted life years (QALY) and life years (LY) gained comparing sacubitril/valsartan versus enalapril and valsartan, respectively. One-way sensitivity analysis using Tornado graphics and a probability sensitivity analysis (using 1000 simulations) were performed. Considering that the instability of the financial system is almost constant, we also ran alternative scenarios considering short-term time horizons (at 3 and 5 year). These time frames were intended to represent changes in healthcare coverage that are a by-product of labour market instability, a characteristic of unstable economies; it implies that the payers implicitly prefer a shorter term perspective given that the coverage provided by an specific payer to an specific employee would change when he/she eventually loses or changes the job, in systems where a substantial percentage of people gets health care access through their insertion in the labour market. Finally, we performed an analysis based on a more conventional 3.5% discount rate for both costs and effects (as it is recommended by NICE) assuming stable financial conditions.

## Results

**Fig. 1:**
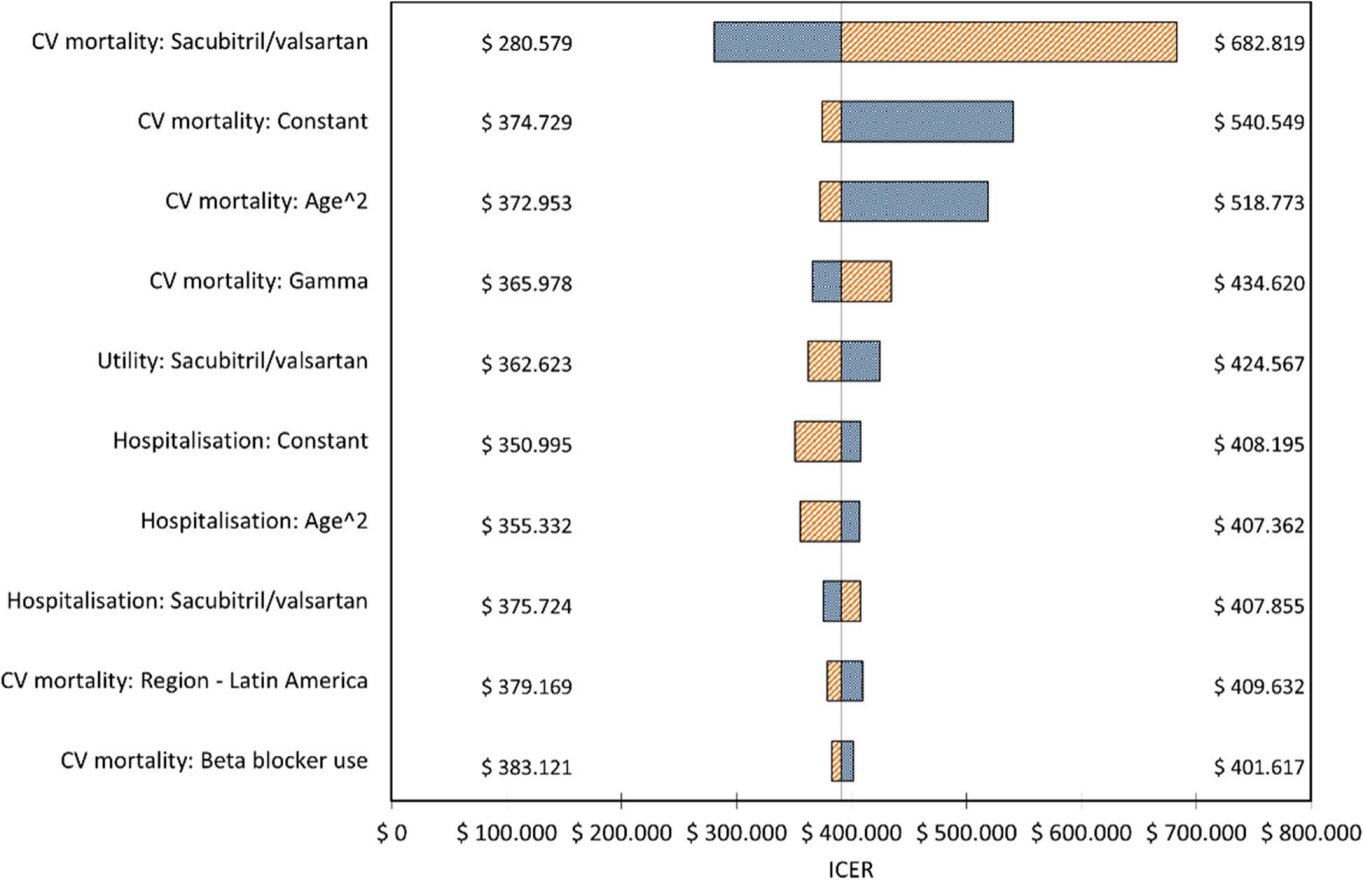
One-way sensitivity analysis (Tornado) for social security health care payer. *ICERs expressed as cost in Argentinian pesos (ARS) per QALY gained. Central line indicates the value of ICER in central estimation of every coefficient in the model; extremes of bars indicate value of ICER in the limits of 95% CI of the coefficients. Blue areas indicate the region of every bar towards lower values of the coefficient under concern; red areas indicate the region of every bar towards higher values of the coefficient being considered*

**Fig. 2:**
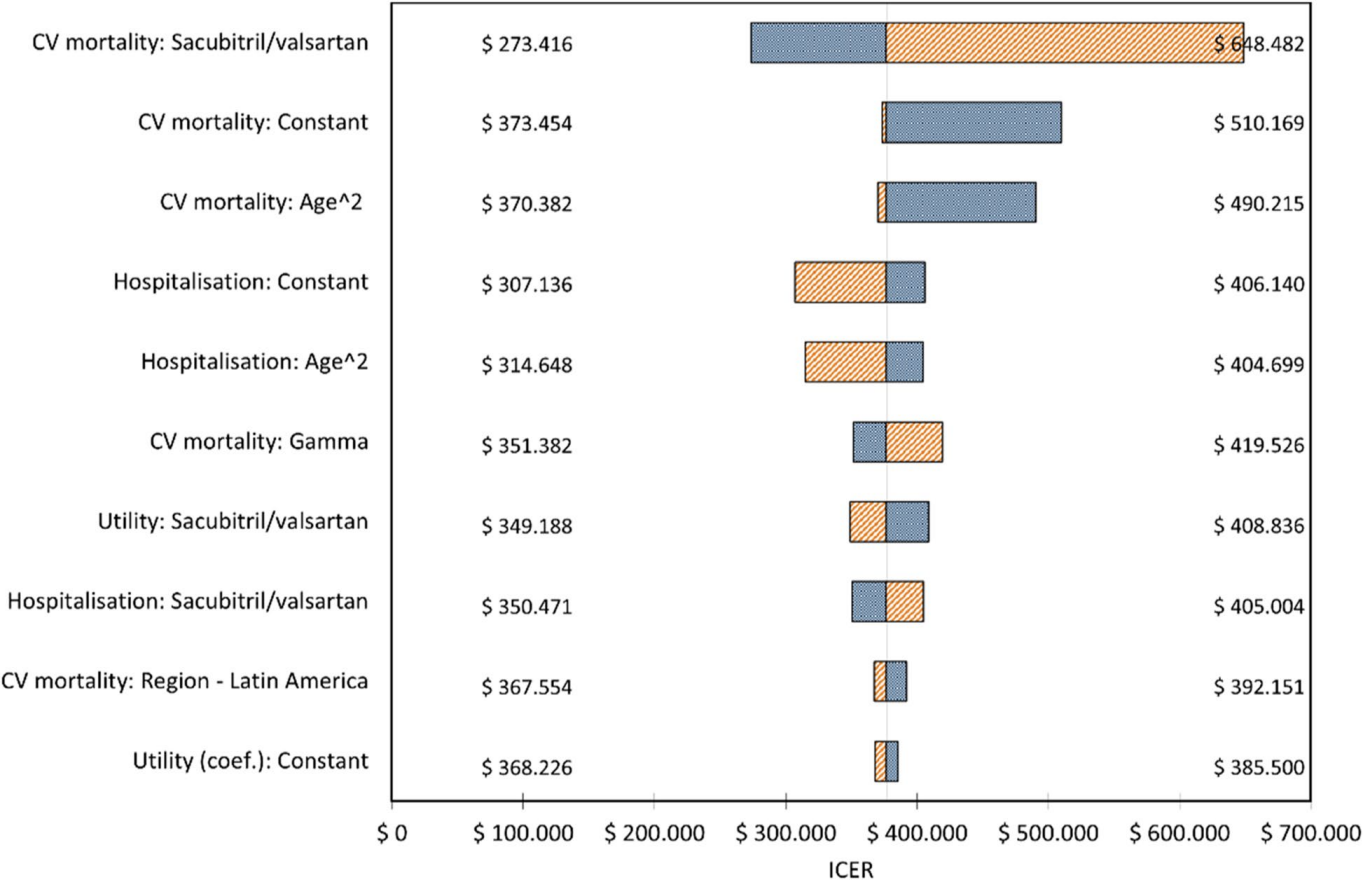
One-way sensitivity analysis (Tornado) for private health care payer. *ICERs expressed as cost in Argentinian pesos (ARS) per QALY gained. Central line indicates the value of ICER in central estimation of every coefficient in the model; extremes of bars indicate value of ICER in the limits of 95% CI of the coefficients. Blue areas indicate the region of every bar towards lower values of the coefficient under concern; red areas indicate the region of every bar towards higher values of the coefficient being considered*

The primary deterministic base case analysis revealed that sacubitril/valsartan was a cost-effective option compared with enalapril, for both payers considered, using a 30-year time horizon, 5% discount rate for effectiveness, 31.64% discount rate for costs and 1 GDP per capita as cost-effectiveness threshold. In the case of the secondary analysis, sacubitril/valsartan was also cost-effective compared with ARBs, considering the same threshold. Table 3 depicts the results of the primary and secondary analyses and showed that the incremental cost-effectiveness ratios for each QALY and LY gained, for both payers, were below the threshold of 520,405.79 ARS. For the primary analysis, the one-way sensitivity analysis depicted in the Tornado graphics (Figs. 1 and 2), which showed that (for both payers considered) the reduction in CV mortality by sacubitril – valsartan was the variable to which the model was more sensitive, followed by a higher baseline cardiovascular mortality and a higher association between age (quadratic term) and CV mortality; other variables showed lower relationship.

**Table 3:** Incremental cost-effectiveness ratios (expressed in QALYs and LYs) at 30-years horizon: sacubitril/valsartan vs enalapril (primary analysis) and vs valsartan (secondary analysis)

**Payer**	**Treatment**	**Total Costs**	**Total QALYs**	**Incremental cost**	**Incremental QALYs**	**ICER**
* Social Security*	enalapril	$165,785	4,45			
	valsartan	$163,631	4,36			
	Sacubitril/valsartan	$355,897	4,94	$190,113	0,49	$391,158
* Private*	enalapril	$276,770	4,45			
	valsartan	$266,033	4,36			
	Sacubitril/valsartan	$459,839	4,94	$183,069	0,49	$376,665
**Payer**	**Treatment**	**Total Costs**	**Total QALYs**	**Incremental cost**	**Incremental LYs**	**ICER**
* Social Security*	enalapril	$165,785	5,61			
	valsartan	$163,631	5,48			
	Sacubitril/valsartan	$355,897	6,16	$190,113	0,55	$345,283
* Private*	enalapril	$276,770	5,61			
	valsartan	$266,033	5,48			
	Sacubitril/valsartan	$459,839	6,16	$183,069	0,55	$ 332,490

*Costs are expressed in Argentinian pesos (ARS). Exchange rate: 1USD* = *89 ARS*

*ICER incremental cost-effectiveness ratio, Cost-effectiveness threshold:* ≤ *1 GDP per capita* = *520,405.79 ARS*

**Fig. 3:**
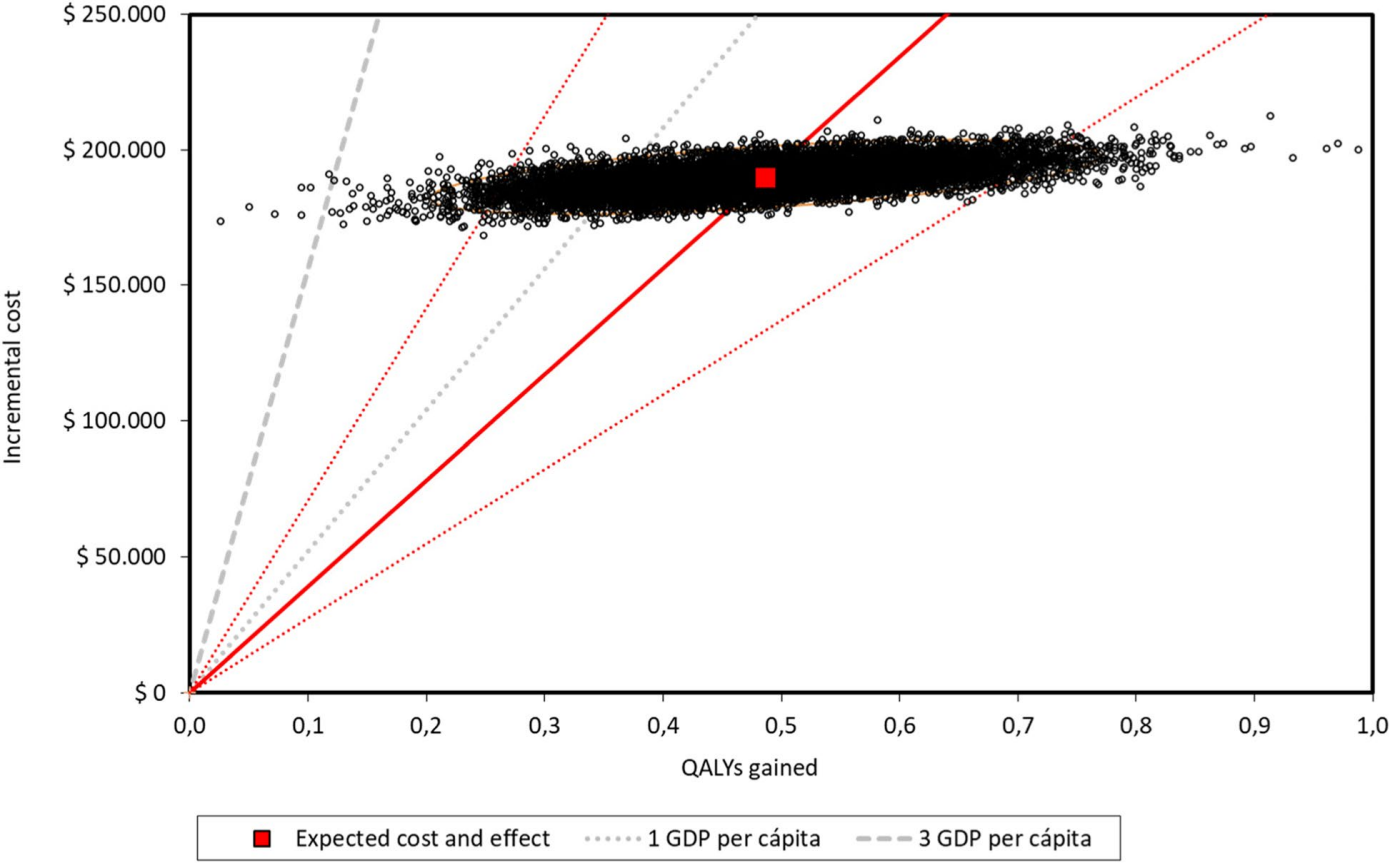
Probabilistic sensitivity analysis and 95% confidence interval ellipse for social security payer (after 1000 simulations) *The red square is the deterministic ICER (cost per for QALY gained). The thick red line represents the probabilistic central estimation with an ICER* = *390,667 ARS per QALY gained The dotted red line, below the thick red one, represents the probabilistic lower 95% confidence limit with an ICER* = *274,242 ARS per QALY gained The dotted red line, above the thick red one, represents the probabilistic upper 95% confidence limit with an ICER* = *708,550 ARS per QALY gained The dotted and hyphened grey lines represent 1 and 3 GDP per capita thresholds, respectively (1 GDP per capita* = *520,405.79 ARS)*

**Fig. 4:**
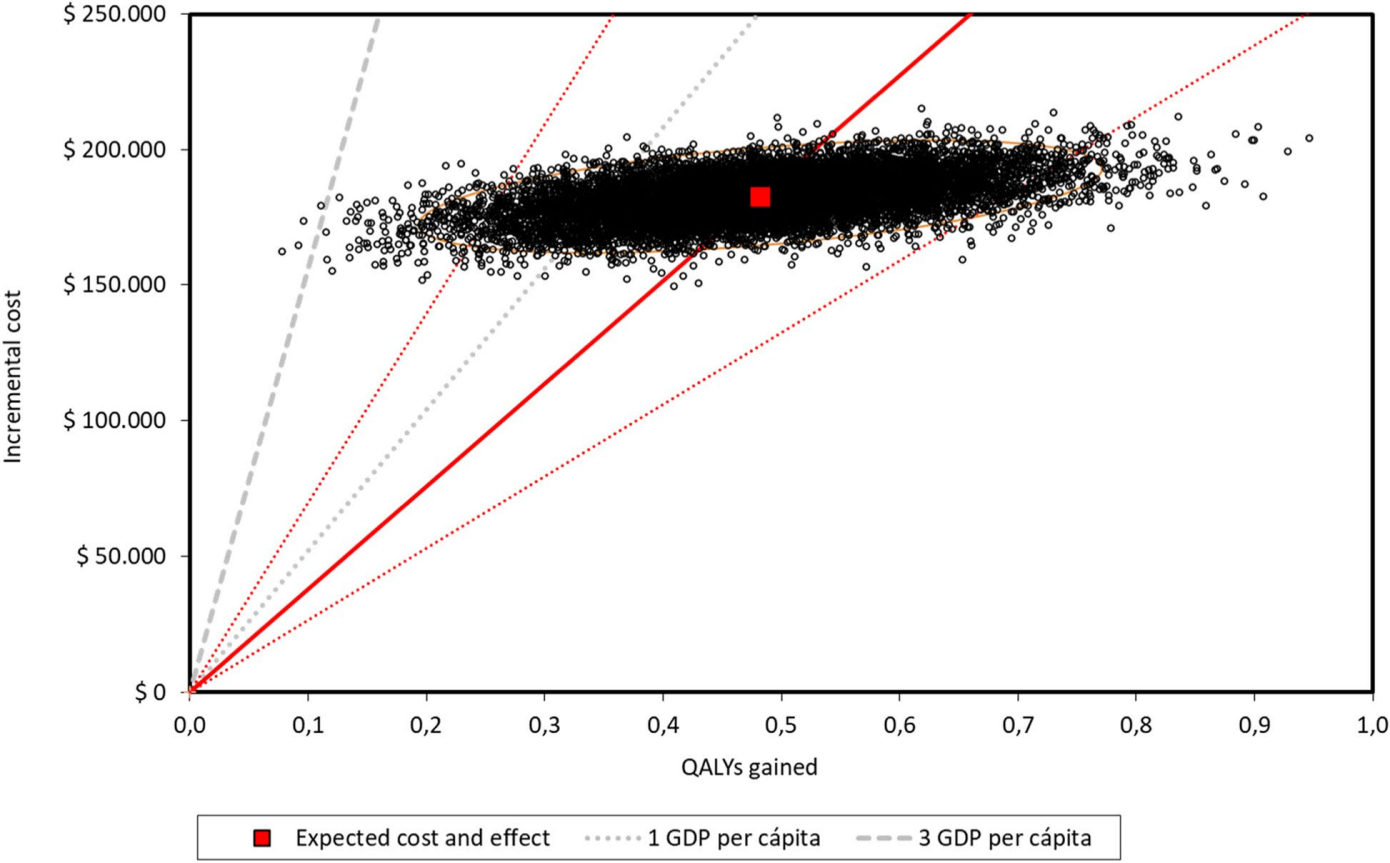
Probabilistic sensitivity analysis and 95% confidence interval ellipse for private payer (after 1000 simulations) *The red square is the deterministic ICER (cost per for QALY gained). The thick red line represents the probabilistic central estimation with an ICER* = *378,882 ARS per QALY gained*, *The dotted red line, below the thick red one, represents the probabilistic lower 95% confidence limit with an ICER* = *265,004 ARS per QALY gained*. *The dotted red line, above the thick red one, represents the probabilistic upper 95% confidence limit with an ICER* = *697,756 ARS per QALY gained*. *The dotted and hyphened grey lines represent 1 and 3 GDP per capita thresholds, respectively (1 GDP per capita* = *520,405.79 ARS)*

**Fig. 5:**
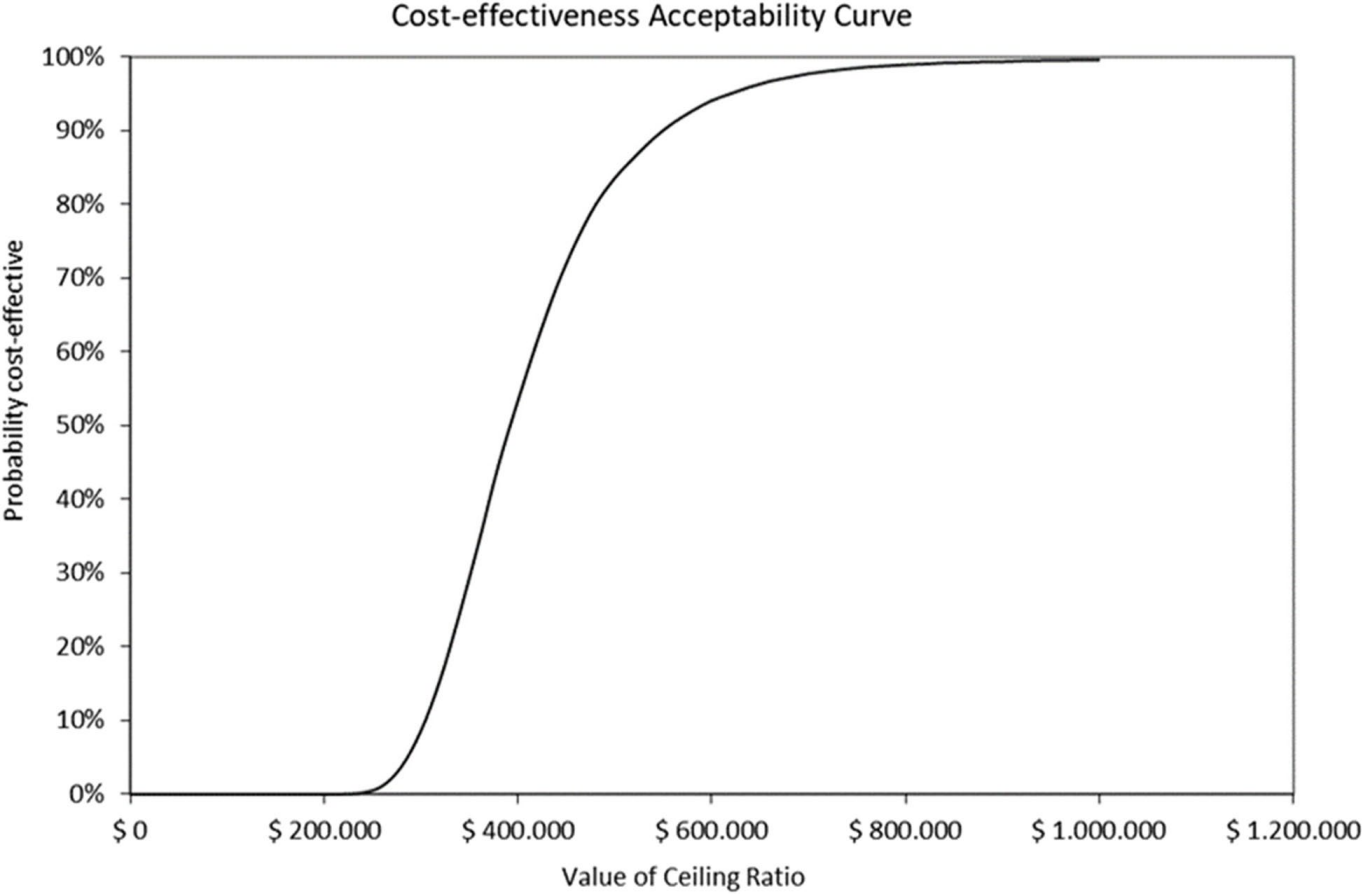
Probability for sacubitril/valsartan of being accepted as cost-effective for a social security payer. *Value of ceiling ratio represents the range of cost-effectiveness thresholds. The curve represents the probability for sacubitril/valsartan of being Accepted as a cost-effective option, considering a range of cost-effectiveness thresholds. At (1 GDP per capita of 520,405.79 ARS the probability is 90%*

**Fig. 6:**
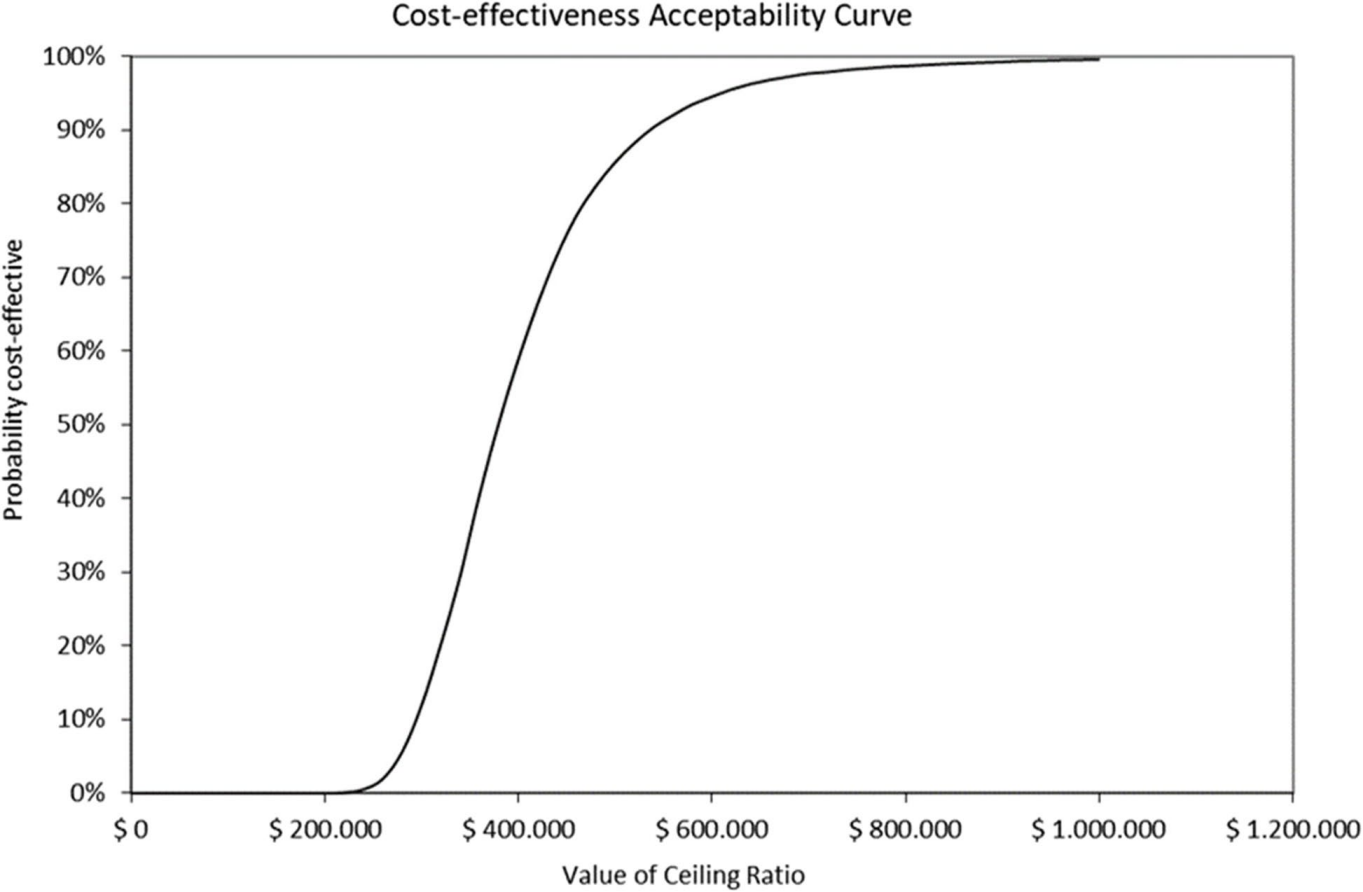
Probability for sacubitril/valsartan of being accepted as cost-effective for a private payer. *Value of ceiling ratio represents the range of cost-effectiveness thresholds. The curve represents the probability for sacubitril/valsartan of being Accepted as a cost-effective option, considering a range of cost-effectiveness thresholds. At (1 GDP per capita of 520,405.79 ARS the probability is 92%*

The probabilistic sensitivity analyses (after 1000 simulations) performed for the primary scenario, in both payers, are depicted in Figs. 3 and 4. Considering the ICERs as cost per QALY gained, the simulations were widely scattered along the axis that represents the clinical effectiveness with low variations in costs. Considering the cost-effectiveness threshold of ≤ 1 GDP per capita it was estimated that the probability for acceptability for sacubitril/valsartan is 86.40% and 88.25% for both social security and private payers, respectively. The acceptability curves were depicted in Figs. 5 and 6.

The short-term time horizon scenarios, set to 3 and 5 years, revealed that sacubitril/valsartan was not a cost-effective option compared with enalapril. For the 3-year scenario, the ICERs per QALY gained were 1,824,383 ARS and 1,737,740 ARS for a social security and a private payer, respectively. For the 5-year scenario the ICERs per QALY gained were 1,143,029 ARS and 1,091,505 ARS for a social security and a private payer, respectively. Analyses for the comparison against valsartan showed similar results. For the 3-year scenario, the ICERs per QALY gained were 1,618,802.01 ARS and 1,612,436.53 ARS for a social security and a private payer, respectively. For the 5-year scenario the ICERs per QALY gained were 988,971 ARS and 988,204 ARS for a social security and a private payer, respectively.

Finally, we ran the analysis using the more conservative scenario of a 3.5% discount rate for both costs and effectiveness in a 30-year horizon. The deterministic analysis revealed an ICER of 857,855 ARS per QALY gained for the comparison of sacubitril/valsartan versus enalapril. That represents a 219% increase in the ICER for the primary analysis compared with the base case analysis.

The full list of scenarios at 3 and 5 years analysed are available in the Supplementary Information.

## Discussion

Our study showed that sacubitril/valsartan, compared with both enalapril and ARBs, was a cost-effective alternative in patients with HFrEF. This result was consistent for both payers considered, in a 30-year time horizon and the local cost-effectiveness threshold. We also showed that the variable to which the model was more sensitive was the efficacy/effectiveness of sacubitril-valsartan: in social security payers, ICER rose to $682.819 in the extreme of lower estimated effect of this drug on mortality (95% CI), whereas in the opposite extreme corresponding to the higher estimated effect, ICER fell to $280.579, with a similar situation in private payers. These results are consistent with the already demonstrated reductions in both mortality and hospitalisations in these patients [3] and with several cost-effectiveness evaluations such in high-income as in low- and middle-income countries [6, 30, 31].

Another relevant point is that In our study we used differential discount rates for health effects and costs to account for the domestic financial setting. This approach to discounting is a controversial issue in health economic analyses. The standard practice to discounting, as stated by NICE, is using the same rate for both costs and health gains, applying figures between 3 to 5% based on the social time preference rate [25, 32]. This methodology has been robust for health technology assessment in countries were social, political, and financial stability is the rule and the projections about the future preferences or consumption of goods and services had a relatively low variability over time: countries with greater GDP per capita and annual GDP per capita growth applied lower discount rates [15]. However, as we stated above, this could not be the most appropriate approach in countries with unstable economies, as is the case for Argentina, with wide variations in its economic and financial variables [33]. As a consequence, we applied a 31.64% discount rate for costs using a widely accepted borrowing rate in Argentina (the BADLAR rate) that is reported by the Central Bank of Argentina [10] and provided a reference of the expected return of investment in Argentina. We also considered, as an alternative (still unrealistic) scenario, the more traditionally used cost discount rate of 3.5%, and as it was expected, it rose the ICER by 119%.

Cost-effectiveness varied across countries due to changes in incidence and severity of the diseases, the availability of health care resources, clinical practice patterns, and relative prices [34]. Therefore, performing an economic evaluation in health care should incorporate jurisdiction-specific data on resource use and cost [14] and this concept could be extended to the financial inputs.

Despite not being officially adopted, the cost-effectiveness threshold in Argentina has been set at ≤ 1 GDP per capita by the National Commission for Health Technology Assessment [27] based on the recommendation of one independent technology assessment agency in the country [35]. This threshold is in the range of the estimation of the opportunity cost for the region (0.5 -1.0 GDP per capita) [36]. Using this threshold, and the opportunity cost of capital approach, the acceptability of sacubitril/valsartan as a cost-effective option was above 85% for both payers considered Considering the alternative discount rate scenario, the intervention was deemed non-cost-effective for the range of thresholds analysed.

Financial instability is a complex phenomenon with diverse causes and consequences. Certainly, the lack of confidence in local currency and its depreciation is one of the many reasons that leads to variation on the economic variables in market economies worldwide. As Inflation is the main consequence of this circumstance, the increase in interest rates and variations in the exchange rate are usual tools used by many central banks in emergent economies to deal with high prices [37]. The impact of this economic environment would undoubtedly affect the expected performance of healthcare systems in stable economies influencing many aspects such as the supply chain [38]. How to deal with all these factors when performing economic evaluations in health care is an open and debatable issue. One approach would be to adjust for inflation. Common modelling practice does not consider inflation because it assumes that there is no inflation rate at all (in other words: keeping constant the price of drugs even in long time horizons), what is counterintuitive given that this assumption has in fact not been true in most countries. Despite financial forecasting is frequently used in most countries, in our case there is an additional difficulty: at least to our knowledge, no reliable tool is available in our country to give estimations of future prices of health technologies. It comes again to the point that unstable economies are characterized by the lack of predictability of most economic variables, what of course includes the future prices of goods and services. As far as inflation rate is increasing worldwide in recent years, the topic has been analyzed by Low et al. opening the debate about the proper way of accounting for inflation rate in the context of NICE reimbursement decisions adjusting for threshold but not for prices of healthcare resources [39]. The other approach could be to adjust for discount rates, and this is the one that we explore in this paper.

Some potential limitations of current study deserve mention. First, it is a randomized clinical trial – based economic evaluation and, as such, prone to lower external validity; in other words, it can be asked if Latin American patients from PARADIGM-HF trial (and their care) that were modelled here are more or less representative of real-world patients cared for heart failure with reduced ejection fraction in Argentina. Nonetheless, there can be no doubt that this kind of economic evaluation has several advantages [40] which have fueled their widespread use. Regarding this model, it deserves mention that this situation of being a randomized clinical trial – based economic evaluation allowed that utilities were obtained from real PARADIGM-HF patients included in Latin America centers, situation that is favourable considering the scarce information about quality of life in our region, where pharmacological modelling is usually made with utilities obtained of sources from other regions. It could also be criticized that our comparators were enalapril and valsartan, only one within the pharmacological class of ACE inhibitors and ARB; however, this issue does not seem relevant considering that several studies have demonstrated a class effect of ACE inhibitors and ARB when used to treat heart failure with reduced ejection fraction, and enalapril was the ACE inhibitor used in the PARADIGM-HF [3]. The events considered in the model did not include other potentially relevant outcomes in this population, such as stroke, atrial fibrillation, cardiac device implantation or other cardiac invasive procedures, but in order to keep the model straightforward we think they should not be included considering that there was no information about the impact of sacubitril-valsartan on them. Finally, the referred ICER threshold in Argentina is in fact only a non-mandatory recommendation by National Commission for Health Technology Assessment, and so it is just a reference useful to put our results in context.

## Conclusion

As heart failure with reduced ejection fraction is the most severe form of HF, the availability of innovative drugs like sacubitril/valsartan that reduced both mortality and hospitalization for HF was an attractive therapeutic alternative to standard care with ACE inhibitors or ARBs and currently recommended therapeutic option in clinical practice guidelines worldwide. It was deemed as a cost-effective treatment in many countries with stable economies, disregarding their income. Our study, designed to assess sacubitril/valsartan in Argentina, highlighted the importance of using domestic inputs for the assessment of this drug (and for the health technology assessment process in general) taking into account the unstable financial environment. This approach should be considered to make more accurate the decision- making process in health care.

## Supplementary Information

**Additional file 1.**

## Data Availability

All data generated or analyzed in this phartmacoeconomic study are included in this submission.

## Abbreviations

ACEI: Angiotensin converting enzyme inhibitors
ARB: Angiotensin receptor blockers
ARNI: Angiotensin receptor-neprilysin inhibitor
ARS: Argentinian pesos
CI: Confidence interval
GDP: Gross domestic product
HF: Heart failure
HFREF: Heart failure with reduced ejection fraction
ICER: Incremental cost-effectiveness ratio
LVEF: Left-ventricular ejection fraction
LY: Life years
NICE: National institute for health care and excellence
QALY: Quality adjusted life-year
