# Supplementary material for: Cost-effectiveness in unstable economies: the case of sacubitril/valsartan in heart failure with reduced ejection fraction in Argentina
Source: Health Econ Rev. 2023 Feb 18;13:13. doi: 10.1186/s13561-023-00427-w (PMC9938575; doi:10.1186/s13561-023-00427-w)
Supplement: Supplementary file 1 — Additional file 1. [file 13561_2023_427_MOESM1_ESM.docx]

Supplementary Information

A. 3-year Scenario

Social Security Payer

SACUBITRIL/VALSARTAN versus ENALAPRIL

| **ICER** | **$ 1824383** |
| --- | --- |

| **COST-EFFECTIVENESS** | ACEi | Sacubitril/valsartan | Incremental |
| --- | --- | --- | --- |
| Total costs (discounted) | $ 114872 | $ 241665 | $ 126793 |
| Total QALYs (discounted) | 2.01 | 2.08 | 0.07 |
| Incremental cost/ QALY | - | - | **$ 1824383,44** |
| Total life years (discounted) | 2.46 | 2.51 | 0.05 |
| Incremental cost/ LYG | - | - | $ 2501196 |
| **COSTS** | ACEi | Sacubitril/valsartan | Incremental |
| Primary therapy | $ 2034 | $ 135784 | $ 133750 |
| Background therapy | $ 13645 | $ 13892 | $ 247 |
| Hospitalisation | $ 77395 | $ 66447 | -$ 10948 |
| HF management | $ 21033 | $ 21414 | $ 381 |
| Adverse events | $ 766 | $ 763 | -$ 3 |
| Titration | $ 0 | $ 3366 | $ 3366 |
| Societal | $ 0 | $ 0 | $ 0 |
| Average annual therapy costs | $ 6383 | $ 59707 | $ 53323 |
| Average annual non-therapy costs | $ 40386 | $ 36695 | -$ 3690 |
|  | | | |
| **OUTCOMES** | ACEi | Sacubitril/valsartan | Incremental |
| HF hospitalisations | 0.21 | 0.19 | -0.03 |
| Other CV hospitalisations | 0.31 | 0.27 | -0.04 |
| Non-CV hospitalisations | 0.32 | 0.27 | -0.04 |
| No. of hospitalisations per year | 0.35 | 0.29 | -0.05 |
| CV mortality (%) at year 2 | 14% | 11% | -0.03 |
| All-cause mortality (%) at year 2 | 17% | 14% | -0.02 |
| Expected survival (years) | 2.63 | 2.68 | 0.06 |

SACUBITRIL/VALSARTAN versus ARB

| **ICER**  **$ 1618802** | | | | |
| --- | --- | --- | --- | --- |
| **COST-EFFECTIVENESS** | ARA II | | Sacubitril/valsartan | Incremental |
| Total costs (discounted) | $ 114063 | | $ 241665 | $ 127602 |
| Total QALYs (discounted) | 2,00 | | 2,08 | 0,08 |
| Incremental cost/ QALY | - | | - | $ 1618802,01 |
| Total life years (discounted) | 2,44 | | 2,51 | 0,06 |
| Incremental cost/ LYG | - | | - | $ 2009087 |
|  | | | | |
| **COSTS** | ARA II | | Sacubitril/valsartan | Incremental |
| Primary therapy | $ 8606 | | $ 135784 | $ 127178 |
| Background therapy | $ 13582 | | $ 13892 | $ 309 |
| Hospitalisation | $ 70192 | | $ 66447 | -$ 3745 |
| HF management | $ 20937 | | $ 21414 | $ 477 |
| Adverse events | $ 746 | | $ 763 | $ 17 |
| Titration | $ 0 | | $ 3366 | $ 3366 |
| Societal | $ 0 | | $ 0 | $ 0 |
| Average annual therapy costs | $ 9081 | | $ 59707 | $ 50626 |
| Average annual non-therapy costs | $ 37602 | | $ 36695 | -$ 907 |
|  | | | | |
| **OUTCOMES** | | ARA II | Sacubitril/valsartan | Incremental |
| HF hospitalisations | | 0.19 | 0.19 | -0.01 |
| Other CV hospitalisations | | 0.28 | 0.27 | -0.01 |
| Non-CV hospitalisations | | 0.29 | 0.27 | -0.01 |
| No. of hospitalisations per year | | 0.31 | 0.29 | -0.02 |
| CV mortality (%) at year 2 | | 14% | 11% | -0.03 |
| All-cause mortality (%) at year 2 | | 17% | 14% | -0.03 |
| Expected survival (years) | | 2.62 | 2.68 | 0.06 |

B. 5-year Scenario

Social Security Payer

SACUBITRIL/VALSARTAN versus ENALAPRIL

| **ICER**  **$ 1143029** | | | |
| --- | --- | --- | --- |
| **COST-EFFECTIVENESS** | ACEi | Sacubitril/valsartan | Incremental |
| Total costs (discounted) | $ 143774 | $ 304319 | $ 160545 |
| Total QALYs (discounted) | 2,89 | 3,04 | 0,14 |
| Incremental cost/ QALY | - | - | **$ 1143028,68** |
| Total life years (discounted) | 3,57 | 3,69 | 0,12 |
| Incremental cost/ LYG | - | - | $ $ 1301258 |
|  | | | |
| **COSTS** | ACEi | Sacubitril/valsartan | Incremental |
| Primary therapy | $ 2.545 | $ 171.484 | $ 168.939 |
| Background therapy | $ 17.078 | $ 17.544 | $ 466 |
| Hospitalisation | $ 96.868 | $ 83.918 | -$ 12.950 |
| HF management | $ 26.325 | $ 27.044 | $ 719 |
| Adverse events | $ 959 | $ 964 | $ 5 |
| Titration | $ 0 | $ 3.366 | $ 3.366 |
| Societal | $ 0 | $ 0 | $ 0 |
| Average annual therapy costs | $ 5.500 | $ 51.208 | $ 45.709 |
| Average annual non-therapy costs | $ 34.796 | $ 31.233 | -$ 3.563 |
|  | | | |
| **OUTCOMES** | ACEi | Sacubitril/valsartan | Incremental |
| HF hospitalisations | 0.33 | 0.28 | -0.04 |
| Other CV hospitalisations | 0.48 | 0.42 | -0.06 |
| Non-CV hospitalisations | 0.48 | 0.42 | -0.06 |
| No. of hospitalisations per year | 0.36 | 0.30 | -0.06 |
| CV mortality (%) at year 2 | 14% | 11% | -0.03 |
| CV mortality (%) at year 5 | 34% | 28% | -0.05 |
| All-cause mortality (%) at year 2 | 17% | 14% | -0.02 |
| All-cause mortality (%) at year 5 | 41% | 35% | -0.05 |
| Expected survival (years) | 3.97 | 4.12 | 0.14 |

SACUBITRIL/VALSARTAN versus ARB

| **ICER**  **$ 988971** | | | |
| --- | --- | --- | --- |
| **COST-EFFECTIVENESS** | ARA II | Sacubitril/valsartan | Incremental |
| Total costs (discounted) | $ 142439 | $ 304319 | $ 161881 |
| Total QALYs (discounted) | 2,88 | 3,04 | 0,16 |
| Incremental cost/ QALY | - | - | **$ 988971,00** |
| Total life years (discounted) | 3,55 | 3,69 | 0,14 |
| Incremental cost/ LYG | - | - | $ 1050087 |
|  | | | |
| **COSTS** | ARA II | Sacubitril/valsartan | Incremental |
| Primary therapy | $ 10747 | $ 171484 | $ 160738 |
| Background therapy | $ 16961 | $ 17544 | $ 583 |
| Hospitalisation | $ 87654 | $ 83918 | -$ 3737 |
| HF management | $ 26145 | $ 27044 | $ 899 |
| Adverse events | $ 932 | $ 964 | $ 32 |
| Titration | $ 0 | $ 3366 | $ 3366 |
| Societal | $ 0 | $ 0 | $ 0 |
| Average annual therapy costs | $ 7833 | $ 51208 | $ 43375 |
| Average annual non-therapy costs | $ 32436 | $ 31233 | -$ 1203 |
|  | | | |
| **OUTCOMES** | ARA II | Sacubitril/valsartan | Incremental |
| HF hospitalisations | 0.29 | 0.28 | -0.01 |
| Other CV hospitalisations | 0.43 | 0.42 | -0.01 |
| Non-CV hospitalisations | 0.43 | 0.42 | -0.01 |
| No. of hospitalisations per year | 0.32 | 0.30 | -0.02 |
| CV mortality (%) at year 2 | 14% | 11% | -0.03 |
| CV mortality (%) at year 5 | 35% | 28% | -0.06 |
| All-cause mortality (%) at year 2 | 17% | 14% | -0.03 |
| All-cause mortality (%) at year 5 | 41% | 35% | -0.06 |
| Expected survival (years) | 3.95 | 4.12 | 0.17 |

C. 3-year Scenario

Private Payer

SACUBITRIL/VALSARTAN versus ENALAPRIL

| **ICER**  **$ 1737740** | | | | | |
| --- | --- | --- | --- | --- | --- |
| **COST-EFFECTIVENESS** | ACEi | | Sacubitril/valsartan | | Incremental |
| Total costs (discounted) | $ 191774 | | $ 312545 | | $ 120771 |
| Total QALYs (discounted) | 2,01 | | 2,08 | | 0,07 |
| Incremental cost/ QALY | - | | - | | **$ 1737739,86** |
| Total life years (discounted) | 2,46 | | 2,51 | | 0,05 |
| Incremental cost/ LYG | - | | - | | $ 2382410 |
|  | | | | | |
| **COSTS** | ACEi | Sacubitril/valsartan | | Incremental | |
| Primary therapy | $ 2034 | $ 135784 | | $ 133750 | |
| Background therapy | $ 13645 | $ 13892 | | $ 247 | |
| Hospitalisation | $ 135867 | $ 116649 | | -$ 19219 | |
| HF management | $ 39155 | $ 39863 | | $ 709 | |
| Adverse events | $ 1073 | $ 1082 | | $ 9 | |
| Titration | $ 0 | $ 5276 | | $ 5276 | |
| Societal | $ 0 | $ 0 | | $ 0 | |
| Average annual therapy costs | $ 6.383 | $ 59707 | | $ 53323 | |
| Average annual non-therapy costs | $ 71696 | $ 64970 | | -$ 6726 | |
|  | | | | | |
| **OUTCOMES** | ACEi | Sacubitril/valsartan | | Incremental | |
| HF hospitalisations | 0.21 | 0.19 | | -0.03 | |
| Other CV hospitalisations | 0.31 | 0.27 | | -0.04 | |
| Non-CV hospitalisations | 0.32 | 0.27 | | -0.04 | |
| No. of hospitalisations per year | 0.35 | 0.29 | | -0.05 | |
| CV mortality (%) at year 2 | 14% | 11% | | -0.03 | |
| All-cause mortality (%) at year 2 | 17% | 14% | | -0.02 | |
| Expected survival (years) | 2.63 | 2.68 | | 0.06 | |

SACUBITRIL/VALSARTAN versus ENALAPRIL

| **ICER**  **$ 1612437** | | | |
| --- | --- | --- | --- |
| **COST-EFFECTIVENESS** | ARA II | Sacubitril/valsartan | Incremental |
| Total costs (discounted) | $ 185445 | $ 312545 | $ 127100 |
| Total QALYs (discounted) | 2,00 | 2,08 | 0,08 |
| Incremental cost/ QALY | - | - | **$ 1612436,53** |
| Total life years (discounted) | 2,44 | 2,51 | 0,06 |
| Incremental cost/ LYG | - | - | $ 2001187 |
|  | | | |
| **COSTS** | ARA II | Sacubitril/valsartan | Incremental |
| Primary therapy | $ 8606 | $ 135784 | $ 127178 |
| Background therapy | $ 13582 | $ 13892 | $ 309 |
| Hospitalisation | $ 123224 | $ 116649 | -$ 6575 |
| HF management | $ 38975 | $ 39863 | $ 888 |
| Adverse events | $ 1058 | $ 1082 | $ 24 |
| Titration | $ 0 | $ 5276 | $ 5276 |
| Societal | $ 0 | $ 0 | $ 0 |
| Average annual therapy costs | $ 9081 | $ 59707 | $ 50626 |
| Average annual non-therapy costs | $ 66817 | $ 64970 | -$ 1847 |
|  | | | |
| **OUTCOMES** | ARA II | Sacubitril/valsartan | Incremental |
| HF hospitalisations | 0.19 | 0.19 | -0.01 |
| Other CV hospitalisations | 0.28 | 0.27 | -0.01 |
| Non-CV hospitalisations | 0.29 | 0.27 | -0.01 |
| No. of hospitalisations per year | 0.31 | 0.29 | -0.02 |
| CV mortality (%) at year 2 | 14% | 11% | -0.03 |
| All-cause mortality (%) at year 2 | 17% | 14% | -0.03 |
| Expected survival (years) | 2.61 | 2.68 | 0.07 |

D. 5-year Scenario

Private Payer

SACUBITRIL/VALSARTAN versus ENALAPRIL

| **ICER**  **$ 1091505** | | | |
| --- | --- | --- | --- |
| **COST-EFFECTIVENESS** | ACEi | Sacubitril/valsartan | Incremental |
| Total costs (discounted) | $ 240025 | $ 393333 | $ 153308 |
| Total QALYs (discounted) | 2,89 | 3,04 | 0,14 |
| Incremental cost/ QALY | - | - | **$ 1091505,25** |
| Total life years (discounted) | 3,57 | 3,69 | 0,12 |
| Incremental cost/ LYG | - | - | $ 1242602 |
|  | | | |
| **COSTS** | ACEi | Sacubitril/valsartan | Incremental |
| Primary therapy | $ 2545 | $ 171484 | $ 168939 |
| Background therapy | $ 17078 | $ 17544 | $ 466 |
| Hospitalisation | $ 170052 | $ 147318 | -$ 22734 |
| HF management | $ 49006 | $ 50344 | $ 1338 |
| Adverse events | $ 1343 | $ 1367 | $ 24 |
| Titration | $ 0 | $ 5276 | $ 5276 |
| Societal | $ 0 | $ 0 | $ 0 |
| Average annual therapy costs | $ 5500 | $ 51208 | $ 45709 |
| Average annual non-therapy costs | $ 61772 | $ 55347 | -$ 6425 |
|  | | | |
| **OUTCOMES** | ACEi | Sacubitril/valsartan | Incremental |
| HF hospitalisations | 0.33 | 0.28 | -0.04 |
| Other CV hospitalisations | 0.48 | 0.42 | -0.06 |
| Non-CV hospitalisations | 0.48 | 0.42 | -0.06 |
| No. of hospitalisations per year | 0.36 | 0.30 | -0.06 |
| CV mortality (%) at year 2 | 14% | 11% | -0.03 |
| CV mortality (%) at year 5 | 34% | 28% | -0.05 |
| All-cause mortality (%) at year 2 | 17% | 14% | -0.02 |
| All-cause mortality (%) at year 5 | 41% | 35% | -0.05 |
| Expected survival (years) | 3.97 | 4.12 | 0.14 |

SACUBITRIL/VALSARTAN versus ARB

| **ICER**  **$ 988204** | | | |
| --- | --- | --- | --- |
| **COST-EFFECTIVENESS** | ARA II | Sacubitril/valsartan | Incremental |
| Total costs (discounted) | $ 231578 | $ 393333 | $ 161755 |
| Total QALYs (discounted) | 2,87 | 3,04 | 0,16 |
| Incremental cost/ QALY | - | - | **$ 988203,95** |
| Total life years (discounted) | 3,54 | 3,69 | 0,15 |
| Incremental cost/ LYG | - | - | $ 1049273 |
|  | | | |
| **COSTS** | ARA II | Sacubitril/valsartan | Incremental |
| Primary therapy | $ 10747 | $ 171484 | $ 160738 |
| Background therapy | $ 16961 | $ 17544 | $ 583 |
| Hospitalisation | $ 153878 | $ 147318 | -$ 6560 |
| HF management | $ 48671 | $ 50344 | $ 1673 |
| Adverse events | $ 1321 | $ 1367 | $ 45 |
| Titration | $ 0 | $ 5276 | $ 5276 |
| Societal | $ 0 | $ 0 | $ 0 |
| Average annual therapy costs | $ 7833 | $ 51208 | $ 43375 |
| Average annual non-therapy costs | $ 57636 | $ 55347 | -$ 2289 |
|  | | | |
| **OUTCOMES** | ARA II | Sacubitril/valsartan | Incremental |
| HF hospitalisations | 0,29 | 0,28 | -0,01 |
| Other CV hospitalisations | 0,43 | 0,42 | -0,01 |
| Non-CV hospitalisations | 0,44 | 0,42 | -0,01 |
| No. of hospitalisations per year | 0,33 | 0,30 | -0,02 |
| CV mortality (%) at year 2 | 14% | 11% | -0,03 |
| CV mortality (%) at year 5 | 35% | 28% | -0,07 |
| All-cause mortality (%) at year 2 | 17% | 14% | -0,03 |
| All-cause mortality (%) at year 5 | 42% | 35% | -0,06 |
| Expected survival (years) | 3,94 | 4,12 | 0,18 |
